# Assessment of natural regeneration of mangrove with reference to edaphic factors and water in Southern Gulf of Kachchh, Gujarat, India

**DOI:** 10.1016/j.heliyon.2019.e02250

**Published:** 2019-08-08

**Authors:** L. Das, R. Patel, H. Salvi, R.D. Kamboj

**Affiliations:** Gujarat Ecological Education and Research Foundation, P.O. Sector-7, Gandhinagar, Gujarat, India

**Keywords:** Ecology, Plant biology

## Abstract

The study was carried out to determine the natural regeneration of four species of mangroves along with estimation of physico-chemical characteristics of sediment and water from seven sites of mangroves in the southern Gulf of Kachchh. Spatial variation of different parameters of water and sediment investigated were: water-pH (7.87–8.04); Salinity (37.07–39.42 ppt); Nitrate (1.21–2.71 ppm); Nitrite (0.03–0.08 ppm); Phosphate (0.39–0.95 ppm) and sediment-pH (7.39–7.61); Bulk density (0.36–0.54 g/cc); Particle density (1.19–1.68 g/cc); Organic carbon (0.77–1.05%); and Organic matter (1.06–1.71%). The density (recruit/sq. m) of natural recruitment of four mangrove species was in order of *Avicennia marina > Ceriops tagal > Aegiceras corniculatum* > *Rhizophora mucronata.* Cluster analysis grouped seven sites in three major clusters *i.e.* Group A (Poshitra & Khijadiya - 91% similarity); Group B (Dedeka-Mundeka, Kalubhar & Pirotan- 94% similarity) and Group C (Sikka & Jodiya- 93% similarity) whereas Non-metric multidimensional scaling showed formation of two groups (Coastal and Islands) depending on the environmental conditions and mangrove natural regeneration. Principal component analysis showed the number of parameters such as salinity, texture and organic carbon which affects the natural regeneration of mangrove species in the study area.

## 1 Introduction

Coastal and marine ecosystems are resilient habitats with high functional diversity. Mangrove forests are the most important coastal tidal ecosystems because of their unique ecological functions, services and socio-economic value to local communities and nations (Jusoff, 2013). Mangroves grow on nutrient-rich, hypoxic, muddy substrates with variations in salinity (Ferreira et al., 2007). Sediments of mangrove are of marine alluvial origin, transported as sediments and deposited by rivers and sea. These sediments are comprised of different percentage of sand, silt and clay. Further, mixture of silt and clay forms mud which are rich in organic matter (Hossain and Nuruddin, 2016). Physico-chemical properties of sediment and water such as particle size fractions (texture), bulk density, particle density, pH, organic matter, salinity and nutrients are major abiotic factors that support the growth of mangroves. These abiotic factors also determine the species composition and structure of mangrove forests (Sherman et al., 1998).

Each plant species has a certain tolerance for each environmental factor and a complex of environmental factors determines the actual distribution of plants in nature (Waring and Major, 1964). Moreover, mangroves are well adapted to natural phenomena and generally recover quickly from both minor and major periodic disturbances through natural regeneration, without the need for planting (Jimenez et al., 1985; Alongi, 2009; Schmitt and Duke, 2016). The major advantage of natural regeneration is that the resulting forest is expected to be more similar to the local mangrove species. In addition, natural regeneration is relatively easy and establish vigorously, less labour is required and result in minimum soil disturbance. However, it may be hampered by lack of seeds and propagules, pollution, poor sediment conditions or disturbed hydrodynamics of the site (Field et al., 1998).

A number of authors (Saha and Choudhury, 1995; Joshi and Ghose, 2003; Marchand et al., 2006; Rao and Rao, 2014; Kiranmai et al., 2015; Ataullah et al., 2017; Agarwal et al., 2016; Trivedi et al., 2016; Biswas et al., 2017) have studied the physical and chemical characteristics of soil and water with reference to mangroves in India. However, less information is available regarding physico-chemical variables of soil and water with special emphasis on mangrove regeneration in India (Saravanakumar et al., 2008; Kathiresan et al., 1996). Hence, the present investigation attempted to record different physio-chemical variables with reference to mangrove regeneration at 7 selected sites of southern Gulf of Kachchh that can indicate the linkage between sediment, water and natural regeneration of mangrove.

## 2 Materials and methods

### 2.1 Study area

The Gulf of Kachchh (GoK) is a wedge like extension of the Arabian Sea which penetrates into the land mass. Geographically it is located at 20° 15′ to 23° 35′ North latitude and 68° 05′ to 70°22′ East longitude on west coast of India. This funnel shaped east-west oriented, seismically active zone provides habitat to a variety of marine flora and fauna. The coastal configuration of the entire Gulf is more or less irregular with a number of islands, creeks and bays. Except for an extensive area from the mouth of the gulf to the center, which consists of rocks (sand stone), the remainder consists of silt and clay with patches of the fine sand (Naik et al., 1991). The area covered by mangroves along the Gujarat coast is the second largest in India, next to the Sundarbans area and major mangroves covered area of the state is confined to the Gulf of Kachchh (Singh et al., 2006).

**Fig. 1:**
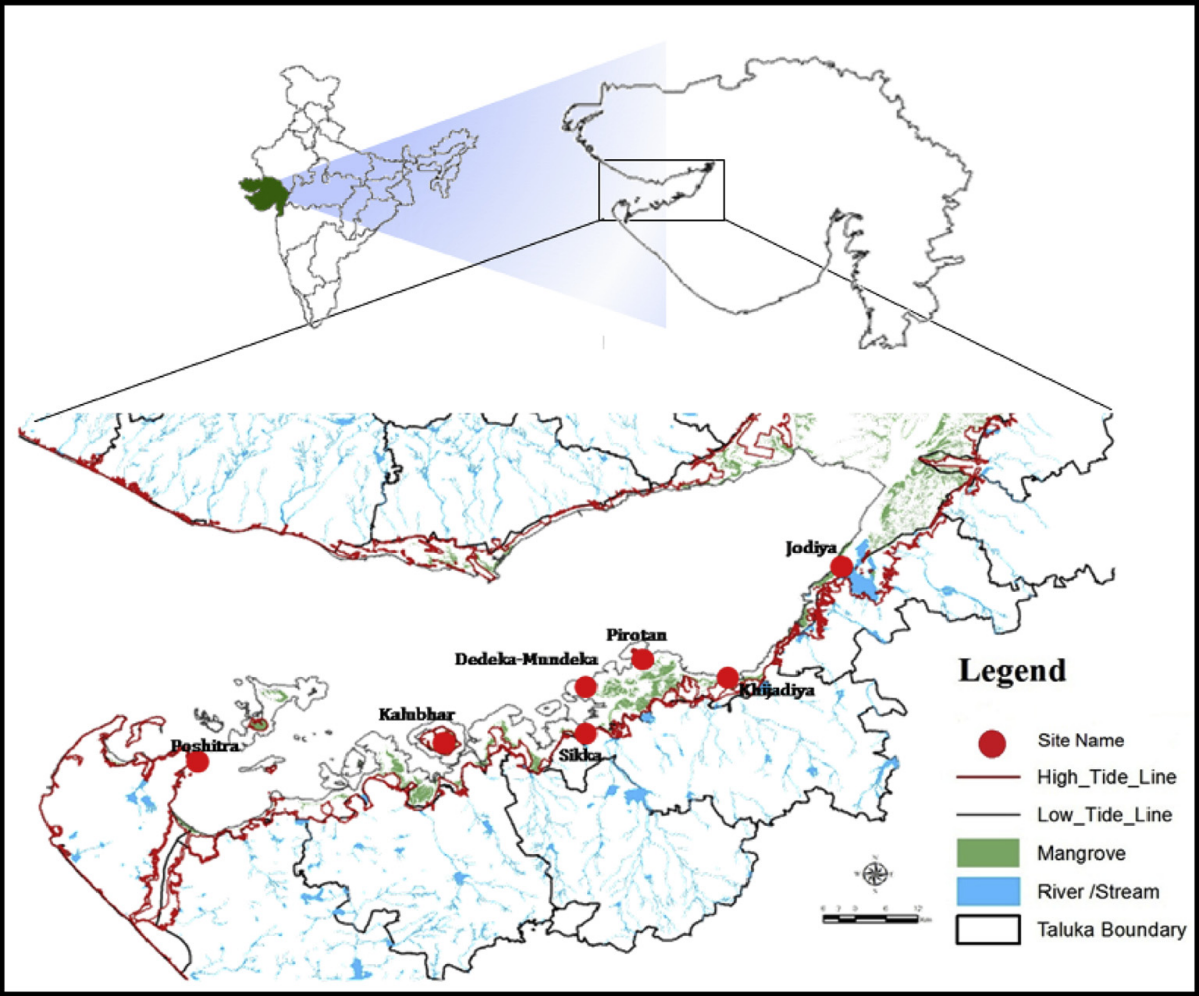
Map showing study area and selected sampling sites of southern Gulf of Kachchh.

To achieve the objective of present study, seven important mangrove sampling sites *viz*; Dedeka-Mundeka, Kalubhar, Pirotan, Poshitra, Sikka, Khijadiya and Jodiya were selected from southern GoK. From these sites, the water and sediment samples were collected and analyzed. Along with this enumeration of natural regeneration of mangrove was also carried out at selected sites which are depicted in Fig. 1 and their GPS locations are given in Table 1.

**Table 1:** Details of seven sample sites of Southern Gulf of Kachchh.

SN	Site name	Island/coastal	GPS coordinates	No. of plots (5 m × 5 m)	No. of quadrates (1 m × 1 m)	Area studied (in ha.)
1	Dedeka-Mundeka (DM)	Island	22° 32′ 39.6″N 69° 52′ 18.9″E	10	50	0.005
2	Kalubhar (KA)	Island	22° 26′ 22.7″N 69° 38′ 48.3″E	10	50	0.005
3	Pirotan (PI)	Island	22° 35′ 44.03″N 69° 57′ 2.04″E	5	25	0.0025
4	Poshitra (PO)	Coastal	22° 28′ 12.4″N 69° 43′ 52.9″E	4	20	0.0020
5	Sikka (SI)	Coastal	22° 49′ 17.7″N 69° 20′ 33.8″E	10	50	0.005
6	Khijadiya (KH)	Coastal	22° 31′ 20.7″N 70° 07′ 54.1″E	10	50	0.005
7	Jodiya (JO)	Coastal	22° 43′ 22.1″N 70° 16′ 46.0″E	30	150	0.015

### 2.2 Field data collection

Field data collection was conducted from November- 2011 to December-2014. To collect the data pertaining to natural regeneration, 1 sq. km. area was randomly selected from mangrove covered area. In each 1 sq. km. area, 5 plots of 5 sq. m were laid randomly and a distance of 100 m was maintained between two adjacent plots. Efforts were made to lay plots in a straight line (transect), but whenever it was not possible to enter the mangroves, the plots were laid 100 m aside. Within each plot, 5 quadrates (1 m × 1 m) were laid (4 quadrates laid at 4 corners and 1 in the center of each plot). Taking into account the average length of propagules, seedling (length 1–50 cm) and sapling (length 51–150 cm) have been considered for enumeration of natural regeneration (Tomlinson, 2004). For the study in the southern Gulf of Kachchh, a total of 7 mangrove covered sites were explored by laying a total of 79 plots (5 m × 5 m size) comprising of 395 quadrates (1 m × 1 m size).

The water samples were collected on monthly basis from November 2010 to December 2014 during high tide whereas the sediment samples were collected during low tide from the sampling sites. The water and sediment pH was measured *in-situ* by using pH pen (make: Eutech) and pH spear (make: Eutech) respectively.

### 2.3 Water and sediment analysis

The salinity was measured from the values of chloride obtained *ex-situ* by Titration method as per standard methods for the examination of water and waste water (Rice et al, 2000). The nutrients *i.e.* Nitrate and Phosphate were estimated by following the standard methodology (Strickland and Parsons, 1972). The sediment samples were collected in zip-lock polythene bags from selected sites on monthly basis. The collected sediment samples were first air dried at room temperature crushed using a porcelain mortar and pestle and then sieved for further analysis (Saha et al., 2001; Dalai et al., 2004). The organic carbon content was determined by following Walkley and Black's method (1934). The moisture content, bulk density and particle density were determined by using the gravimetric method (Maiti, 2003). The average values of water and sediment parameters were used to perform various statistical analyses to interrelate it with mangrove regeneration.

### 2.4 Statistical analysis

Various multivariate statistical analyses including Principal Component Analysis (PCA), non-metric multidimensional scaling (NMDS) and cluster analysis (CA) were performed using Paleontological Statistics Software Package (PAST version 2.17c) in order to know the relation between studied environmental parameters and natural regeneration of mangroves.

## 3 Results and discussion

### 3.1 Natural regeneration status of mangroves

In the present study, density of natural recruitment of four mangrove species *viz. Avicennia marina (AM)*, *Rhizophora mucronata (RM)*, *Ceriops tagal (CT)* and *Aegiceras corniculatum (AC)* was recorded from 7 sites of southern GoK. Regeneration of *A. marina* was found in all the studied sites. The regeneration of *R. mucronata*, *C. tagal* and *A. corniculatum* was only recorded in three islands of southern GoK which might have attributed to lower salinity at Island sites as compared to coastal sampling sites. In the 7 sites of Southern GoK, the average values of density along with standard deviation of natural recruitment of all the mangrove species was found to be 50.05 ± 5.26 recruits/sq m for seedling and 17.69 ± 1.56 recruits/sq. m for sapling, the ratio of seedlings to saplings being 1:0.35. At species level, *A. marina* shows the highest density of natural recruitments (66.86 ± 6.76 recruits/sq. m) followed by *C. tagal* (0.62 ± 0.096 recruits/sq. m), *A. corniculatum* (0.16 ± 0.018 recruits/sq. m) and *R. mucronata* (0.10 ± 0.014 recruits/sq. m) (Table 2).

**Table 2:** Status of density (recruits./Sq.m) of natural recruitment in the 7 sites of the Southern GoK.

Sites/species		*AM*		*RM*		*CT*		*AC*		SDG to SPG ratio		
		SDG	SPG	SDG	SPG	SDG	SPG	SDG	SPG			
Islands	Dedeka- Mundeka	4	3.14	0.02	–	–	–	–	–	1:0.78	1:0.62	1:0.35
	Kalubhar	1.98	0.88	–	–	0.28	0.26	0.04	0.04	1:0.51		
	Pirotan	2.6	1.12	0.04	0.04	0.04	0.04	0.04	0.04	1:0.46		
Coastal sites	Poshitra	25.75	7	–	–	–	–	–	–	1:0.27	1:0.30	
	Sikka	11.66	3.92	–	–	–	–	–	–	1:0.34		
	Khijadiya	0.22	0.11	–	–	–	–	–	–	1:0.50		
	Jodiya	3.38	1.1	–	–	–	–	–	–	1:0.32		

Note: AM: *Avicennia marina*, AC: *Aegiceras corniculatum*, CT: *Ceriops tagal*, RM: *Rhizophora mucronata*, SDG: Seedlings and SPG: Saplings.

The present observation of the highest density of natural recruitments of *A. marina* is supported by Pandey and Pandey (2009), who observed that the natural recruitments of *A. marina* was the highest (22.92 recruits/sq. m) in selected mangrove habitats of South Gujarat, whereas *A. corniculatum* and *C. tagal* shows 0.12 natural recruits/sq m and 0.014 natural recruits/sq. m, respectively. The natural recruitment in Sundarban mangrove areas of Bangladesh in respect of *A. corniculatum* and *C. tagal* reported 1002 recruits/ha and 53 recruits/ha, respectively in between 2010 to 2016 (Rahman, 2017). According to Mchenga and Ali (2014), in general, the difference in regeneration between one species and another depends on different factors such as type of soil and seed structure.

It was calculated that density of natural recruitments of all the four species in the 3 islands was 14.6 ± 1.13individual/sq m [Seedling (SDG) 9.04 ± 1.34 recruits/sq m and sapling (SPG) 5.56 ± 0.92 recruits/sq m] whereas it was 53.14 ± 5.04 recruits/sq. m (SDG 41.01 ± 6.85 recruits/sq m and SPG 12.13 ± 1.93 recruits/sq m) in the 4 coastal sites. The seedling to sapling ratio in the 3 islands was 1:0.62 and in the 4 coastal sites it was 1:0.30. The present study revealed that the numbers of recruits per sq. m. i.e. density is the maximum in the 4 coastal sites although the seedling to sapling ratio was the highest in the 3 islands of Southern GoK.

The density of natural recruitment of *A. marina* was found to be the highest in Poshitra 32.75 recruits/sq m (seedling 25.75 recruits/sq. m & sapling 7 recruits/sq. m) whereas the seedling to sapling ratio was the lowest *i.e.* 1:0.27. On the other hand the minimum density of natural recruitment of *A. marina* was found in Khijadiya 0.33 recruits/sq m (seedling 0.22 recruits/sq. m & sapling- 0.11 recruits/sq. m) and the seedling to sapling ratio was 1:0.50. Pirotan exhibits the maximum density of natural recruitments of *Ceriops tagal* 0.08 recruits/sq m (seedling – 0.04 recruits/sq. m & sapling- 0.04 recruits./sq. m) and *Rhizophora mucronata* 0.08 recruits sq m (seedling 0.04 recruits/sq. m & sapling 0.04 recruits./sq. m). The maximum density of natural recruitments of *Aegiceras corniculatum* was found both in Pirotan and Kalubhar 0.08 recruits/sq m (seedling 0.04 recruits/sq. m & sapling 0.04 recruits./sq. m).

### 3.2 Physico-chemical parameters of water and sediment

#### 3.2.1 Water

**Fig. 2:**
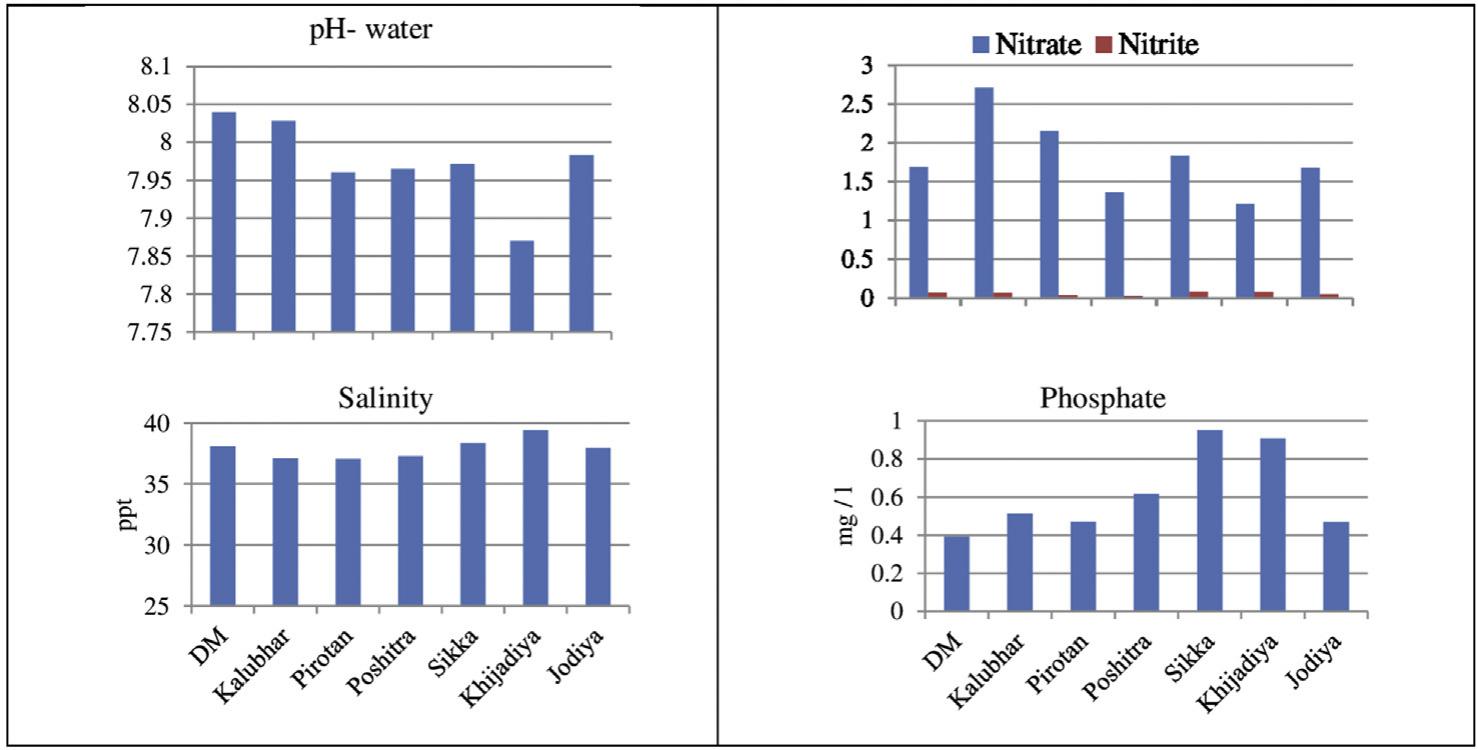
Graphs showing variation in selected water quality parameters at seven sites of GoK.

The pH of water showed alkaline nature throughout the study period. The highest and the lowest value of pH were recorded at Dedeka- Mundeka (8.04) and Khijadiya (7.87), respectively. Satpathy et al. (2009) also observed a pH range of 7.7–8.3 along the coastal waters of Kalpakkam, South east coast of India. The salinity of water varied between 37.06 ppt and 39.42 ppt and the maximum and minimum values were observed at Khijadiya and Pirotan, respectively. Kunte et al. (2003) stated that large variation of air and water temperature and scanty rainfall makes the GoK a high saline water (50 ppt) body. The result also corroborates with the findings of Devi et al. (2014) where the salinity was recorded in the range of 33.4 ppt–43.8 ppt at Vadinar, Gulf of Kachchh. There occurs spatial variation in nutrient content among the studied sites. In mangrove ecosystem, nutrients are considered as the most important parameters that influence growth, reproduction and metabolic activities of biotic components. The distribution of nutrients is mainly based on season, tidal conditions and fresh water influx from land (Saravanakumar et al*.* 2008). The highest concentration of Nitrate was recorded at Kalubhar (2.71 mg/l) whereas the lowest was found at Khijadiya (1.21 mg/l). Natural water has low nitrite concentration because bacteria quickly convert Nitrite to other more stable nitrogen ions. The nitrite concentration ranged between 0.03 mg/l to 0.08 mg/l with the lowest and the highest values at Poshitra and Sikka, respectively. The values of phosphate ranged between 0.30 mg/l and 0.95 mg/l; the highest values was recorded at Sikka whereas the lowest at Dedeka- Mundeka (Fig. 2). The variation may be due to the various processes like adsorption and desorption of phosphates and buffering action of sediments under varying environmental conditions (Rajasegar, 2003).

#### 3.2.2 Sediment

**Fig. 3:**
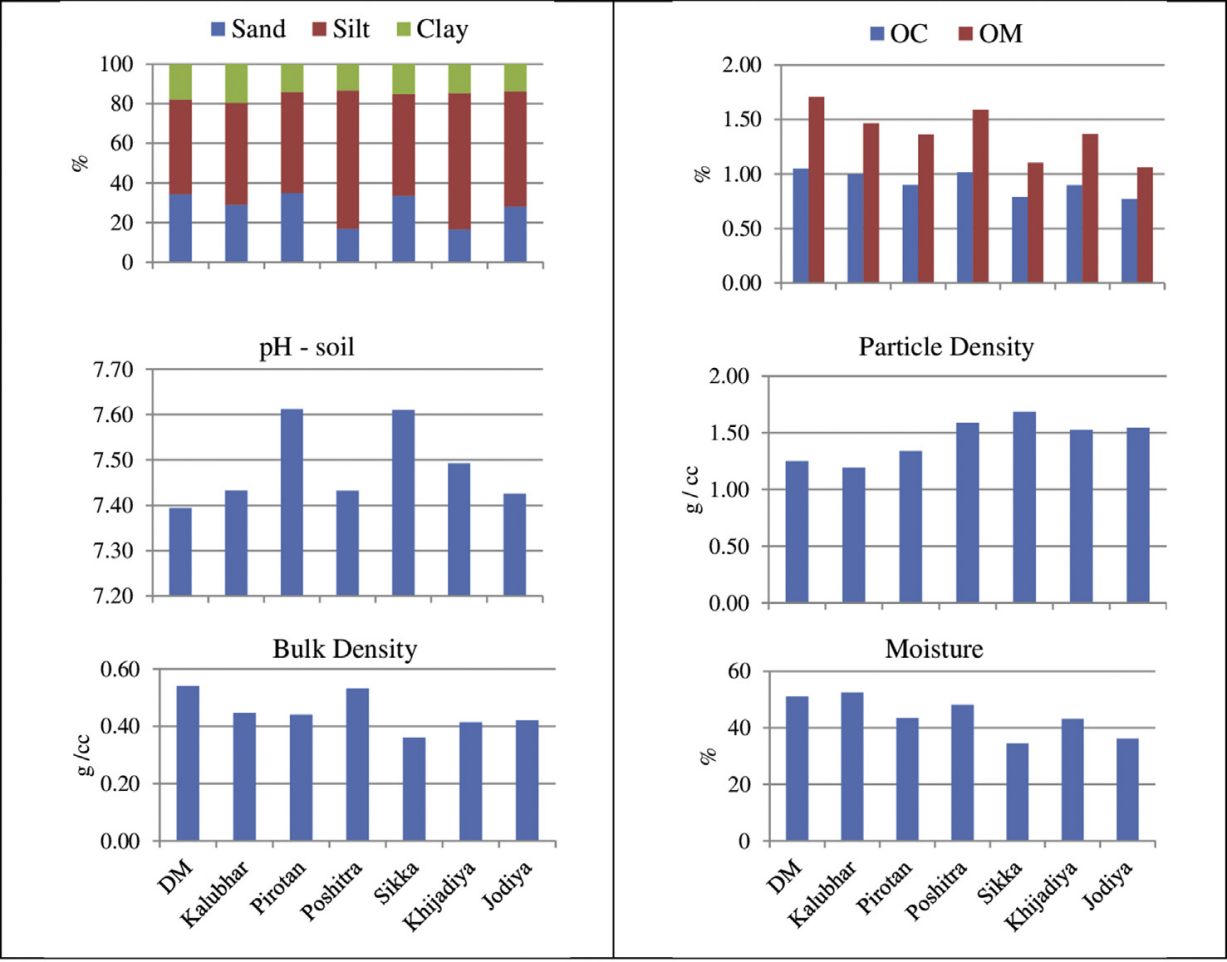
Graph showing variation in selected sediment quality parameters at seven sites of GoK.

Table 4 shows the physico-chemical properties of sediment of different mangrove forests worldwide. The percentage of sand, silt and clay varied among different sites. In present study, the highest and the lowest percentage of sand were recorded at Pirotan (35.0%) and Khijadiya (16.5%), respectively. On the other hand, the silt content varied from 70.0% to 50.9% at Poshitra and Pirotan, correspondingly; whereas clay has the maximum value at Kalubhar (19.9%) and the minimum value at Poshitra (13.2%). Overall, the substratum was mainly composed of silt with admixture of sand and clay. Therefore, by and large, the sediment texture was found to be silty loam at all the selected sites. Mangrove forests are areas having low-energy water environment which is conducive for the sedimentation of clay particles. However, sediments with higher percentage of sand have also been reported to have mangroves (Hossain and Nuruddin, 2016). Similar to water, the pH of sediment falls between 7.39 and 7.61; the greatest value was recorded at Pirotan whereas the least was recorded at Dedeka-Mundeka (Fig. 3). Mangrove sediments are mostly alkaline as reported by various authors (Tam et al., 1995; Tam and Wong, 1998). However, many other workers have recorded acidic pH in mangrove sediment which might be attributed to the oxidation of FeSO_4_ and FeS to H_2_SO_4_ (Holmer et al., 1994). Sediment acidity may also have resulted from decomposition of mangrove litter (Lacerda et al., 1995). The values of bulk density ranged from 0.36 g/cc to 0.54 g/cc with the lowest and the highest values recorded at Sikka and Dedeka- Mundeka respectively. As organic matter increases, bulk density decreases. Variable values of bulk density were reported by various authors in the mangrove forests of world (Table 4): As compared to other studies, the average bulk density of the sediments in Gulf of Kachchh is quite low showing less water content within the soil stratum. It is also affected by the sedimentation. The wave action that carry sediments and deposit on the coast, play vital role in sediment composition and compaction. Main source of soil organic carbon and matter is litter. Soil organic carbon determines the nature of the soil composition. Sandy soil holds very less organic matter as compared to the clay soil. Sites which are coasts have invariably sandy beaches and thus sediment of such sites is less in organic carbon and organic matter content. In present study, the amount of Organic Carbon (OC) and Organic Matter (OM) was found to be the maximum at Dedeka-Mundeka (OC = 1.05% & OM = 1.71%) and the minimum at Jodiya (OC = 0.77% & OM = 1.06%). As depicted in Table 4, the OC and OM contents of the mangrove sediments varied widely all over the world. The lower values of OC and OM were reported from mangrove sediments of Indian region which indicates the poor nutritional conditions of the mangrove forest (Hossain and Nuruddin, 2016). Particle density showed the highest value at Sikka (1.64 g/cc) and the lowest value at Kalubhar (1.19 g/cc). The moisture content values varied between 34.44% to 52.45% at Sikka and Kalubhar, respectively (Fig. 3.). It is a major controlling factor for many hydrological processes, especially runoff generation, soil evaporation and plant transpiration.

**Table 3:** Factor matrix obtained by the method of principal components analysis for 7 sites of southern GoK.

Parameters	PC1	PC2	PC3	PC4	PC5
pH- sediment	−0.327	−0.656	−0.404	0.216	0.152
Sand	0.469	−0.682	−0.168	−0.494	0.160
Silt	−0.598	0.640	−0.018	0.439	−0.137
Clay	0.661	−0.294	0.658	−0.067	−0.118
BD	0.518	0.807	−0.124	−0.211	0.145
OC	0.668	0.646	0.157	0.115	0.162
OM	0.584	0.687	0.113	0.050	0.297
PD	−0.921	0.036	−0.156	−0.138	−0.285
Moisture	0.771	0.524	0.211	0.246	0.111
AM	−0.196	0.574	−0.325	−0.207	−0.494
AC	0.681	−0.419	−0.271	0.535	−0.031
RM	0.341	−0.285	−0.692	0.121	0.548
CT	0.690	−0.246	0.297	0.461	−0.399
pH-Water	0.769	−0.064	0.046	−0.561	−0.295
Salinity	−0.692	0.002	0.580	−0.034	0.426
Nitrate	0.748	−0.569	−0.010	0.170	−0.296
Nitrite	−0.158	−0.419	0.845	−0.101	0.202
Phosphate	−0.759	−0.176	0.333	0.268	−0.073
% of Variance	38.582	24.376	14.749	8.955	7.870
Cumulative % of variance	38.582	62.958	71.913	80.869	88.739

Values more than 0.5 (either + or -) are underlined because they are statistically significant.

**Table 4:** Physico-chemical properties of soil of mangrove forests worldwide.

Names of forest	Regions	Soil parameters									
		Sand (%)	Silt (%)	Clay (%)	Soil texture	Bulk density	pH	Moisture	Particle density	Organic carbon (%)	Organic matter (%)
*Avicennia* forest in Apar nature reserve*	East Kalimantan, Indonesia	30.04	39.86	30.10	Clay loam	110.5 (g/100 mL)	4.82	–	–	3.96	6.81
*Ceriops* forest in Apar nature reserve*	East Kalimantan, Indonesia	31.18	35.77	32.05	Clay loam	138.5 (g/100 mL)	3.95	–	–	11.40	19.61
Calabar mangrove swamp*	Nigeria	34.66	44.20	21.14	Clay loam	0.73 (g/cc)	4.80	–	–	6.43	11.06
Hooker Bay mangrove*	SanAndres Island, Colombia	53.17	27.80	18.98	Sandy clay loam	0.9 (g/cc)	6.14	–	–	13.31	22.89
Prentice Island mangrove*	Sunderbans, India	8.10	61.90	30.00	Silty clay	1.07 (g/cc)	8.00	–	–	0.55	0.95
Lotihan Island mangrove *	Sunderbans, India	19.90	40.20	39.90	Silty clay	1.07 (g/cc)	7.50	–	–	0.62	1.07
Sagar Island mangrove*	Sunderbans, India	48.00	36.10	15.90	Silt loam	1.42 (g/cc)	7.40	–	–	0.65	1.12
Harinbari Island mangrove*	Sunderbans, India	24.20	45.80	29.90	Silty clay	1.26 (g/cc)	7.60	–	–	0.75	1.29
Cheringa mangrove*	Bangladesh	9.00	44.00	47.00	Silty clay	1.02 (g/cc)	3.20	–	–	2.92	5.02
Wildlife Sanctuary Sibuti mangrove*	Miri, Sarawak, Malaysia	–	–	–	–	–	3.34	–	–	12.18	20.96
Awat-Awat Lawas mangrove*	Limbang, Sarawak, Malaysia	–	–	–	–	–	3.19	–	–	9.38	16.20
Sundarban mangrove*	NE coast of Bay of Bengal, India	–	–	–	–	–	8.22	–	–		
Sundarban mangrove*	Bangladesh	–	–	–	–	–	7.67	–	–	0.38	0.65
Crumahu river mangrove*	Sao Paulo, Brazil	–	–	–	–	–	6.40	–	–		–
Sundarban Mangrove^#^	Bangladesh	–	–	–	–	–	7.34	25.70 %	–	0.83	–
Tamilnadu Mangrove^@^	Muthupet, Tamilnadu, India	–	–		Silty loam	1.34 (g/cc)	8.31	–	2.27 (g/cc)	0.29	0.59
Pondhichery mangrove^##^	Pondichery coast, India	63.42	21.43	15.14	Sandy clay loam	–	–	–	–	–	2.79
Gulf of Kachchh Mangrove (Present study)	Southern Gulf of Kachchh	27.60	57.00	15.68	Silty loam	0.45 (g/cc)	7.49	44.10 %	1.45 (g/cc)	0.92	1.38

*Hossain and Nuruddin (2016); #Md. Ataullah et al., (2017); @ Vaijayanthi and Vijayakumar 2014; ##Satheeshkumar and Khan (2011).

### 3.3 Linking regeneration patterns to sediment and water parameters

#### 3.3.1 Multivariate statistical analysis

##### 3.3.1.1 Cluster analysis (CA)

**Fig. 4:**
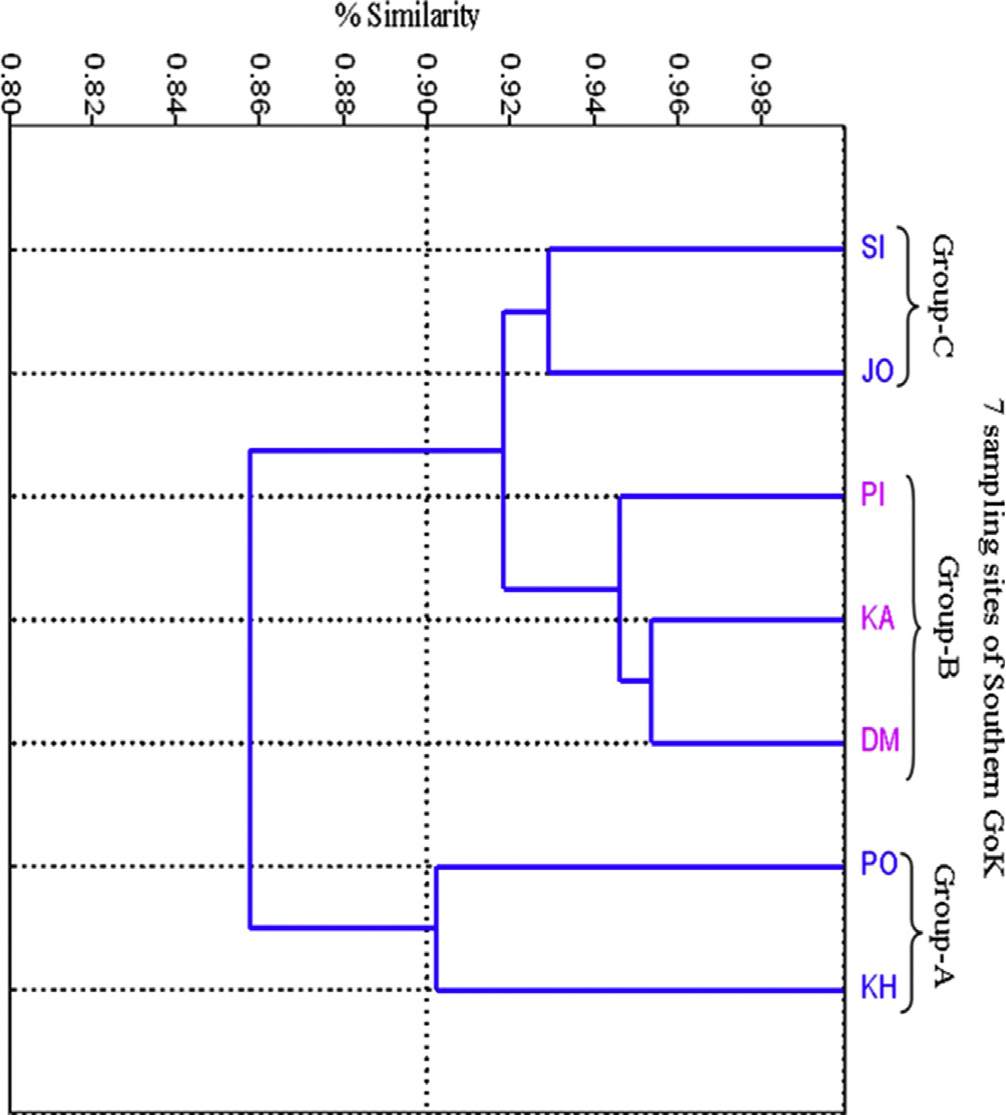
Bray-curties -similarity index on the basis of sediment quality, water quality and mangrove regeneration.

CA was used to detect similar groups between the sampling sites during the study period. CA calculated the physico-chemical characteristics of water and sediment along with regeneration status of Mangrove species and the results are depicted in Fig. 4 showing a dendrogram. The dendrogram was generated using Bray-curtis similarity index and showed three groups. Group-A has included two sites namely Poshitra and Khijadiya with 91% of similarity. Group-B was formulated by three sites *i.e.* Dedeka- Mundeka, Kalubhar and Pirotan with 94% of similarity. Group-C included two coastal sites *i.e.* Jodiya and Sikka showing 93% of similarity. The sites in first group are coastal sites which have less anthropogenic pressures; one being important coral reef area and another a bird sanctuary. Sites in second group are Islands present in GoK and the environmental conditions prevailing there are more or less same. While, the third group is formed by coastal sites again *i.e.* Jodiya and Sikka but, the difference lies in the influence of anthropogenic pressure and activities performed therein. Jodiya is located towards the head region of GoK and is a fish landing center & minor port (Dixit et al., 2010; Kumar et al., 2017) whereas Sikka, located adjacent to Vadinar, has a state-owned thermal power plant, a cement factory, a private jetty, an extensive pipeline network for unloading crude oil and exporting petroleum products, a liquid cargo jetty and another crude oil tank farm located along its coast (Sukumaran et al., 2013).

##### 3.3.1.2 Non-metric multidimensional scaling (NMDS)

**Fig. 5:**
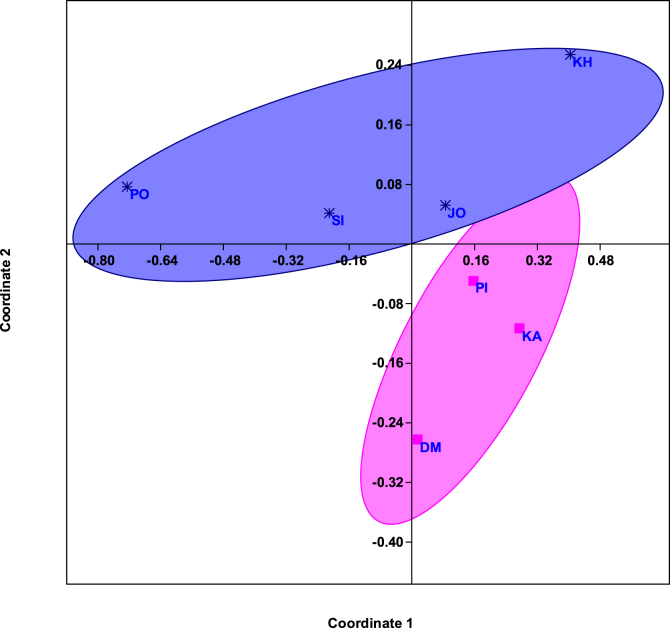
Horn- Similarity measure- Non Metric Multidimentional Scaling. [PO: Poshitra, DM: Dedeka-Mundeka, KA: Kalubhar; PI: Pirotan; SI:Sikka; JO: Jodiya; KH: Khijadiya].

The present study also used NMDS to find out the degree of similarity among the selected sites of southern GoK. The mangrove regeneration, water quality and sediment quality parameters were used to perform this test. Fig. 5 depicts that, the sample points lying close to each other have more similarity in mangrove species composition while, sample plots lying apart from each other showed dissimilarity. The NMDS plot also revealed two groups; one is formed by coastal sites *i.e.* Poshitra, Sikka, Jodiya and Khijadiya whereas another is showing presence of three island sites *i.e.* Dedeka- Mundeka, Kalubhar and Pirotan. These groups also demonstrate the mangrove species composition for natural regeneration which is more at Island sites. As shown in Table 2, DM showed regeneration of two species (*Avicenia marina & Rhizophora mucronata*), Kalubhar has three species (*Ceriops tagal, Aegiceras corniculatum & Avicenia marina* and Pirotan has maximum, *i.e.*, four species (*Avicenia marina, Ceriops tagal, Aegiceras corniculatum and Rhizophora mucronata*) whereas *Avicenia marina* is the only species found to be present at all the coastal sites. Furthermore, it recorded higher regeneration at all the sites indicating its tolerance for wide range of fluctuations in the environmental conditions.

##### 3.3.1.3 Principal Component Analysis (PCA)

**Fig. 6:**
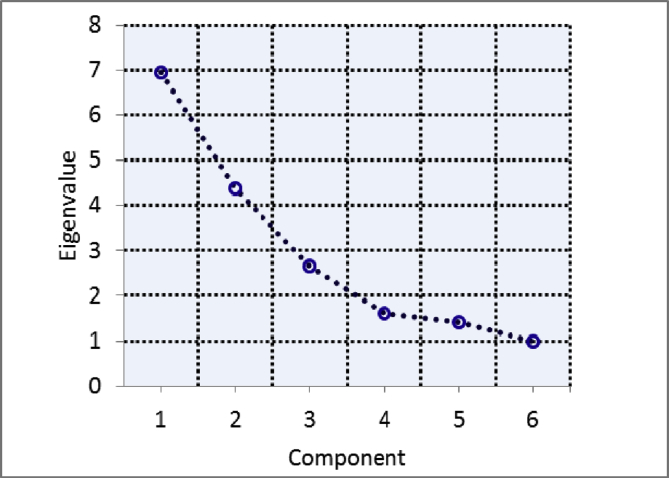
Scree plot between Principal Component and eigenvalue.

PCA is one of the most appropriate and widely used statistical techniques that simplify the large dataset of different variables. The data of various environmental parameters and mangrove regeneration had been applied in statistical software to perform PCA. The first five factors having eigenvalue greater than 1 were chosen for PCA (Fig. 6). Table 3 summarizes the PCA results including the loading, % of variance and cumulative % of variance. It can be observed from the table that the first Principal Component (PC1) included a substantial part of the variables connected positively to Clay, OC, OM, Moisture, *A. marina*, *C. tagal*, pH- water, and Nitrate and negatively to Silt, PD, Salinity and Phosphate. PC2 explained 24.376% of the total variance and showed strong positive loading of Silt, BD, OC, OM, Moisture and *A. marina* whereas negative loading of sediment-pH and sand had been observed. PC3 has strong loading on clay, salinity and nitrite with positive values and on *R. mucronata* with negative value having 14.749% of variance. PC4 has strong loading on *A. corniculatum*, explaining 8.955% of total variance whereas PC5 explained 7.870% of variance with strong loading on *R. mucronata*.

**Fig. 7:**
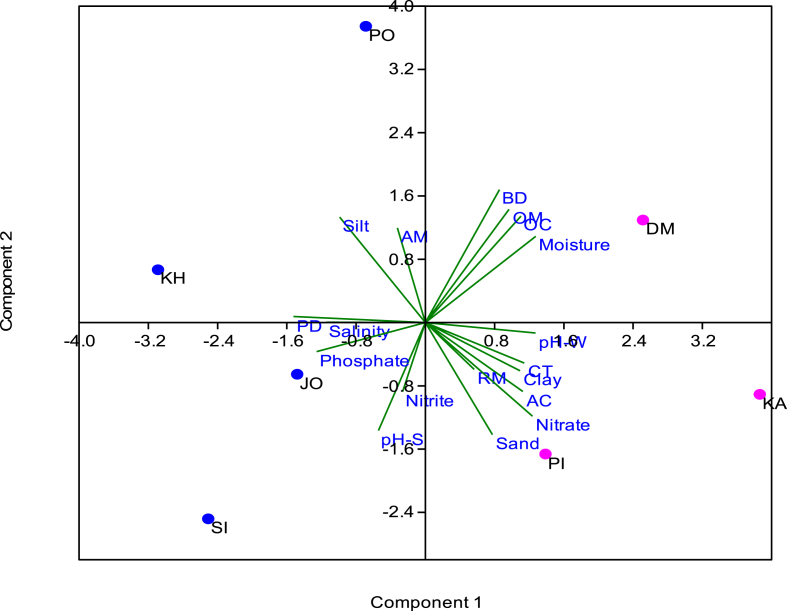
PCA biplot showing the components using environmental parameters and mangrove regeneration data. [pH-S: pH-sediment; BD: Bulk Density; OC: Organic Carbon; OM: Organic Matter; PD: Particle Density; Mo.: Moisture Content; AM: *Avicenia marina*; AC: *Aegiceras corniculatum*; RM: *Rhizophora mucronata*; CT: *Ceriops tagal*; pH-W: pH- water; Sal.: Salinity; NO_3_^-^:Nitrate; NO_2_^-^:Nitrite; PO_4_^3-^:Phosphate, PO: Poshitra, DM: Dedeka-Mundeka, KA: Kalubhar; PI: Pirotan; SI:Sikka; JO: Jodiya; KH: Khijadiya].

Moreover, the PCA biplot (Fig. 7) showed that Dedeka-Mundeka experienced maximum variation in sediment quality parameters *viz;* bulk density, organic matter, organic carbon and moisture. The remaining two island sites experienced variation in water-pH and sediment texture i.e. sand & clay along with natural regeneration of mangrove species, namely, *R. mucronata*, *C. tagal*, and *A. corniculatum*. Coastal sites of Jodiya and Sikka experienced variation in terms of water parameters such as Nitrite, Salinity, Phosphate and sedimentl-pH. The natural regeneration of A*. marina*, silt and particle density varied greatly at Poshitra and Khijadiya.

## 4 Conclusion

The present study provides the information regarding mangrove natural regeneration and its environment (i.e. edaphic factors and water quality) on the basis of multivariate statistical techniques. The study revealed variation in environmental parameters at coastal and island sites which is clearly indicated by cluster analysis and non-multidimentional scaling plot. Moreover, natural recruitment of *A. marina* was higher at all the sites showing its wide range of tolerance to varied environmental conditions whereas, natural recruitment of other three species have been recorded at islands only which could be due to their ability to thrive in lower salinity gradients. Additionally, in present study the values of organic carbon and organic matter were less as compared to other mangrove soils of world which shows reduced nutritional concentration in some mangrove soils of southern GoK. This information would be useful in management decisions of mangrove plantation such as the selection of species and sites that can provide suitable environment for growth of the four mangrove species studied in Gulf of Kachchh. Further, island sites may be consider for plantation of *C. tagal*, *A. corniculatum* and *R. mucronata* as it has lower salinity compared to other coastal areas of GoK. Overall this study specify that sediment and water quality are the factors which can determines the survival and distribution of natural regeneration of mangrove species along the islands and coastal areas of Gulf of Kachchh.

## Declarations

### Author contribution statement

Das L., Patel R.: Performed the experiments; Analyzed and interpreted the data; Contributed reagents, materials, analysis tools or data; Wrote the paper.

Salvi H.: Conceived and designed the experiments.

Kamboj R.D.: Analyzed and interpreted the data.

### Funding statement

This work was supported by the World Bank, Ministry of Environment, Forest and Climate Change (MoEF and CC), National Project Management Unit- Society of Integrated Coastal Management (NPMU-SICOM), and State Project Management Unit- Gujarat Ecology Commission (SPMU-GEC) for providing the financial assistance for this work under the Integrated Coastal Zone Management Project.

### Competing interest statement

The authors declare no conflict of interest.

### Additional information

No additional information is available for this paper.
